# The Impact of Radioiodine (131I) Therapy of Thyroid Disease on Salivary Glands Function and Inflammation: A Comprehensive Review

**DOI:** 10.3390/biomedicines13061404

**Published:** 2025-06-07

**Authors:** Pietro Bellini, Francesco Dondi, Carlo Cappelli, Elisa Gatta, Davide Lombardi, Claudio Casella, Riccardo Morandi, Gianluca Viganò, Luca Camoni, Michela Cossandi, Valentina Zilioli, Francesco Bertagna

**Affiliations:** 1 Nuclear Medicine, ASST Spedali Civili di Brescia, 25123 Brescia, Italy; francesco.dondi@unibs.it (F.D.); luca.camoni@unibs.it (L.C.); michela.cossandi@asst-spedalicivili.it (M.C.); valentina.zilioli@asst-spedalicivili.it (V.Z.); francesco.bertagna@unibs.it (F.B.); 2 Nuclear Medicine, Università degli Studi di Brescia, 25123 Brescia, Italy; 3 Department of Clinical and Experimental Sciences, SSD Endocrinologia, Università degli Studi di Brescia and ASST Spedali Civili di Brescia, 25123 Brescia, Italy; carlo.cappelli@unibs.it (C.C.); elisa.gatta@unibs.it (E.G.); 4 Centro per la Diagnosi e Cura delle Neoplasie Endocrine e delle Malattie della Tiroide, Università degli Studi di Brescia, 25121 Brescia, Italy; 5 Otorhinolaryngology-Head and Neck Surgery, ASST Spedali Civili di Brescia, 25123 Brescia, Italy; davinter@libero.it; 6 Surgical Clinic, Università degli Studi di Brescia and ASST Spedali Civili di Brescia, 25123 Brescia, Italy; claudio.casella@unibs.it (C.C.); rik.morandi@gmail.com (R.M.); 7 Clinical Engineering, ASST Spedali Civili di Brescia, 25123 Brescia, Italy; gianluca.vigano@asst-spedalicivili.it

**Keywords:** thyroid cancer, salivary glands, radioiodine, side effects, xerostomy, sialadenitis

## Abstract

Radioactive iodine therapy has been a well-established treatment for various thyroid conditions since the 1940s, targeting both benign diseases and malignancies. Treatment for benign conditions typically involves low doses of 131I, often requiring no more than two treatments, with the dose either fixed or personalized based on thyroid tissue mass and iodine uptake. In contrast, differentiated thyroid cancer treatment often requires higher doses and multiple administrations, especially for metastatic cases. Recent guidelines and studies have proposed more conservative management strategies, including careful follow-up, due to concerns over the high risk–benefit ratio in selected cases with a low risk of disease recrudescence. Despite its possible efficacy, radioiodine therapy is associated with dose-dependent side effects, the most common of which is salivary gland dysfunction or inflammation, affecting approximately 30% of adult patients. These effects pose significant challenges in nuclear medicine practice. This review aims to summarize the latest evidence on the incidence, impact on quality of life, prevention strategies and the role of these side effects in the decision-making process regarding RAI therapy.

## 1. Introduction

Radioactive iodine (RAI) therapy is a well-established treatment for several thyroid conditions, performed since the 1940s [1]. The targets of RAI therapy include benign diseases such as multinodular goiter (MNG) and Graves’ disease (GD), as well as malignancies, particularly but not only differentiated thyroid cancer (DTC) [2,3,4].

The treatment of benign conditions typically necessitates a low dose of RAI [5,6], with the possibility of choosing between a fixed or personalized dose calculated by estimating the mass of thyroid tissue, typically no more than a few dozen grams, and the time function of iodine uptake in this mass. In addition, the therapy of benign thyroid diseases generally requires no more than two treatments [7,8]. Conversely, the therapy of DTC mandates a higher dose of RAI activity with the administration of at least 1.1 Gigabecquerel (GBq). Furthermore, multiple high-dose RAI administrations are sometimes required, especially in metastatic disease [9,10].

Recently, some international guidelines and studies have proposed the limitation of RAI therapy and the endorsement of a more conservative management approach, based on follow-up, particularly in the treatment of DTC. This is due to the presumed high risk–benefit ratio of the therapy in selected cases following thyroidectomy [11,12], during follow-up in the event of biochemical evidence of disease relapse and/or indeterminate data [13,14] and after a high cumulative dose of RAI [15]. In this context, it has been highlighted that RAI therapy is associated with some dose-dependent side effects, of which salivary gland dysfunction and/or inflammation is one of the most common, occurring in around 30% of adult patients [16]. Although other rare adverse effects such as male infertility and acute leukemia have been reported with high-dose RAI [11,17], xerostomy and sialadenitis may also occur with intermediate-dose RAI and represent a challenge in the daily practice of nuclear medicine [18].

The aim of this comprehensive review is to summarize the most recent evidence on the incidence, impact on quality of life, possible prevention and the possible impact on the decision whether or not to perform RAI therapy due to the possible resulting salivary gland effects. Finally, a schedule will be advanced as a possible reference for mitigating salivary gland toxicity in nuclear medicine clinical practice.

## 2. Mechanism of the Salivary Gland’s Damage and Pre-Clinical Studies

The biological distribution of 131I is mainly due to two different pharmacokinetic mechanisms: Firstly, the elimination route of radioiodine, which is mainly through the kidneys, bladder and urine. Secondly, the different expression of the sodium–iodine symport (NIS) in different organs [19,20], which can allow the concentration of iodine in the cells with an active transport. The thyroid gland, particularly the follicular thyroid cells, present the highest level of NIS expression due to the necessity of the iodine for thyroid hormone production. In any case, NIS is also highly expressed in other tissues such as the mammary glands during lactation, the stomach and the principal salivary glands, particularly the parotid and submaxillary glands, both located in the neck and with a weight of a few grams [21].

Due to this heterogeneous distribution [22], the thyroid is not the only target organ of the radiation derived from RAI: in particular, salivary glands concentrate 131I through the high expression of NIS and result as one of the organs with the highest dose exposure [23]. However, this dose appears to be limited in the treatment of benign thyroid disease, and usually, the mean absorbed doses by these organs are about 0.63 Gray (Gy) for patients with Graves’ disease and 1.1 Gy for multinodular goiter [24]. Instead, in the case of RAI therapy for DTC, the absorbed dose in the salivary glands appears to be very variable and generally higher than 0.2 milligray/megabecquerel (mGy/MBq) for the parotid glands and 0.14 mGy/MBq for the submaxillary glands [25,26], resulting in a higher total absorbed dose depending on the amount of administered RAI activity. As in the thyroid tissue, low-penetration β-particles emitted by RAI (max energy 606 Kilo-electronvolt (keV), mean energy 190 keV) into tissues possess the capacity to ionize the surrounding atoms. This process subsequently results in the damage of cellular deoxyribonucleic acid (DNA), ultimately leading to the demise of the affected cells. If thyroid cells’ death represents the goal of the therapy in both the treatment of DCT and hyperthyroidism, the damage to salivary gland cells is a known and relatively frequent adverse effect resulting in subsequent xerostomia and/or possible sialadenitis [27].

### Pre-Clinical Studies

The mechanisms related to genetic damage derived from RAI have been described in some studies in rats, with possible involvement of the ADAMTS genes, particularly with increased expression of ADAMTS2 and ADAMTS5 and decreased expression of ADAMTS12 [28,29]. Moreover, a single study described that the p53 gene does not appear to be involved in the process [30].

Considering the prevention of salivary gland damage, a number of potential protective agents, including alpha-lipoic acid, curcumin, keratinocyte growth factor-1 (KGF1) and ginseng, have been studied in murine models [31,32,33,34]. In particular, the group of researchers of Kim JM et al. wrote three papers [31,33,34] in which they demonstrated that use of KGF1, curcumin and red ginseng of Korea apparently may guarantee the protection of salivary glands in mice treated with RAI. Furthermore, in a single study on rabbit amifostine it apparently could reduce the risk of salivary gland damage after a high dose of RAI [35].

While murine studies demonstrate the protective effects of KGF1 and curcumin, translational challenges exist. For instance, rat salivary glands exhibit higher NIS expression than humans, and radiation sensitivity varies across species. Human trials are needed to confirm these findings. Future research should prioritize clinical validation of pre-clinical agents like KGF1, adjusting for interspecies metabolic differences [28,29,30,31,32,33,34,35].

## 3. Damage Diagnosis and Assessment

Despite the recognized potential for salivary gland damage to occur subsequently to RAI, the diagnostic process and the quantification of xerostomy grade and its impact on quality of life remain under debate. The evaluation of xerostomy and/or salivary gland inflammation, with the exception of patients enrolled in study protocols, is typically initiated only in instances where patients who have undergone RAI report symptoms. In other scenarios, these findings may be incidentally found during routine ultrasonographic examinations, particularly in the context of thyroid cancer surveillance [36,37].

The diagnosis and subsequent assessment of the patient can be performed by clinical evaluation and diagnostic imaging examinations, including radiological and nuclear medicine procedures. Table 1 at the end of this section summarizes the key characteristics of each method.

### 3.1. Clinical Assessment

The clinical assessment of salivary gland impairment is typically performed by physical examination and the administration of specific questionnaires. In particular, different studies have applied this procedure, with the first method including the evaluation of the presence of specifics signs such as edema, redness in the area of the salivary glands, purulent discharge, cracking at the corners of the mouth, salivary glands atrophy, etc. [38].

The second instrument evaluated both symptoms and the impact on quality of life [39,40]. For example, Buchholzer S. et al. [39] proposed a six-question questionnaire (RAI-6) that assessed the degree of xerostomia, the frequency of salivary gland tingling and swelling, and the impact on quality of life, and produced a score related to the degree of impairment. The score obtained showed a statistical correlation with the administration of RAI therapy and the dose of RAI administered. Another study of Moreddu E. et al. [41] tested a self-administered questionnaire to check for the presence of pain, dry mouth or xerostomia, and discomfort or swelling. The use of the instrument was limited to checking the presence of these side effects after RAI therapy. Although these instruments appear easy to use clinically, they are limited by the subjective perception of symptoms by patients.

### 3.2. Ultrasound Assessment

Salivary gland damage can also be detected by ultrasound [42,43], which is usually performed as part of routine follow-up in thyroid cancer. Despite its convenience and availability, the examination has the limitation that ultrasound changes in the salivary glands sometimes appear before clinical manifestation: a proportion of patients could have a positive ultrasound without any clinical complaint [44]. Regarding ultrasound features, there is general consensus about the correlation between atrophy and xerostomia, and most of the studies focusing on this topic describe a reduced echogenicity and irregular margins in the parotid glands of patients treated with RAI [36,43,44]. There is literature consensus about the correlation between the dose of administered RAI and the probability and severity of these ultrasound findings [36,42]. A quite recent meta-analysis by Lima GAS et al. [45] summarizes these findings; in particular, echotexture heterogeneity was found with a significant difference in patients treated with 131I.

### 3.3. Nuclear Medicine Assessment

Nuclear medicine assessment of salivary gland damage after RAI therapy is not routinely performed in clinical practice. However, in the context of scientific research, it has been described that salivary gland scintigraphy with 99mTc-pertecnetate (99mTc-O4-) can assess the difference in salivary gland function before and after RAI administration, with the possibility of quantification [46,47,48,49]. The most recent of these studies was performed by Wu JQ et al. [49], which described a complete loss of the maximum secretion rate of the parotid glands in the case of treatment with 131I doses higher than 600 millicurie (mCi), but no significant decrease in them with doses lower than 150 mCi. As with the ultrasound findings, the scintigraphic assessment confirms the dose-dependency of the damage, the possible utility of sialagogue drugs/substances and the preferential involvement of the parotid glands. Another analogy with ultrasound findings is the possibility of detecting mild salivary gland damage without clinical manifestations [47].

### 3.4. Other Imaging Techniques

Computed tomography (CT) and magnetic resonance imaging (MRI) are described in a few studies as potentially useful techniques in the diagnosis of salivary gland damage after RAI therapy. In particular, Shen J et al. [50] described a correlation between symptoms and MRI findings. Furthermore, with regard to CT imaging, Nabaa B et al. [51] found that a reduction in salivary gland volume and an increase in attenuation on CT could be indicators of the degree of RAI-induced dysfunction. This technique does not have any practical application in everyday clinical practice.

## 4. Xerostomy and Sialadenitis in Radioiodine Therapy of Benign Thyroid Disease

Treatment of benign thyroid disease, such as hyperthyroidism in Graves’ disease or a hyperfunctioning multinodular goiter, usually requires low doses of RAI: the administered activity is usually between 370 and 555 MBq (10–15 mCi) and rarely, only in the case of a large goiter, exceeds the limit of 1000 MBq [52,53]. Consequently, as reported in the literature, the absorbed dose in the salivary glands appears to be low [26] and the possibility of RAI-induced damage to the salivary glands is very rare, and only occurs with the administration of the higher activity [52]. The risk of sialadenitis and xerostomy appeared to be so negligible that more than one set of international guidelines regulating RAI treatment for benign thyroid disease does not mention these side effects in their documents [54,55]. Concerning single studies, Chielens L et al. [56] described a single centre experience in treating Basedow with RAI in adolescents: in 14 patients treated with doses ranging between 5.8 and 15 mCi, only one presented with a transient salivary gland inflammation (7.14%). Furthermore, Okkalides D [57] reported similar results with only one hyperthyroid patient out of 16 treated with RAI showing persistent salivary gland dysfunction (6.25%). The patient in this study received activities ranging from 360 to 550 MBq. The author also pointed out that a negative psychology of the patients could influence their results negatively. Finally, Ford H et al. [58] carried out a study of salivary composition in patients with hyperthyroidism compared with euthyroid patients. As part of the study design, salivary flow (milliliters per minutes [mL/min]) was also assessed before and after treatment with 370 MBq of 131I in 26 hyperthyroid patients. The results showed a reduction in salivary flow of only 3.1% after RAI therapy, with no statistical difference between flow before and after treatment. These results confirm that xerostomia is a very rare finding in hyperthyroid patients treated with RAI. Anyway, one significant limitation of the reported studies was the small size of the sample groups.

In terms of international guidelines, the American Thyroid Association (ATA) provided its in 2016 [54], representing an example of the lack of indication in this setting. Instead, the European Association of Nuclear Medicine (EANM) published a recent version of its guidelines in 2023 [52], which reported a possible incidence of salivary gland side effects of 20–30%, especially in patients receiving more than 1 Gbq, but considering references about RAI treatment in DTC. Furthermore, the EANM 2023 guideline suggests the administration of lemon juice starting 24 h after treatment as a valid prevention.

## 5. Xerostomy and Sialadenitis in Radioiodine Therapy of DTC

Acute sialadenitis is a known challenge and a common side effect of RAI performed for thyroid cancer treatment. In addition, the possibility of chronic damage, particularly to the parotid glands, with the development of xerostomia, is well known. These effects are generally related to the administered dose and the number of treatments received by the patient [59]. A large number of studies and reviews have been carried out and are available in the literature: the topic will be treated by distinguishing between a single dose of RAI with the aim of remnant ablation and adjuvant intent and the high-dose therapy and/or multiple treatments performed in the case of structural disease due to DTC.

### 5.1. Remnant Ablation and Adjuvant Therapy

Radioiodine therapy in DTC always has the aim of remnant ablation: in this setting, patients generally receive RAI activity between 1.1 and 1.85 GBq (30–50 mCi), as suggested by international guidelines [11,60]. In the presence of adjuvant intent, particularly in patients with an intermediate and high risk of disease recurrence, the administration of RAI activity could be increased up to 3.7–5.55 GBq (100–150 mCi). In general, higher activity is limited to patients with suspected or certain persistence of structural disease.

With regard to the effects of a single dose of RAI on salivary gland inflammation and function, the literature is consistent in concluding that these depend on the activity administered; in particular, lower activities are associated with a low risk of sialadenitis and xerostomia [42,47,49,61,62,63,64], and these symptoms, if they occur, are in most cases transient [50,65,66].

Considering single studies, Horvath et al. [42], evaluating the possible salivary gland impairment by ultrasound examination, did not find any injury in 156 patients who received 1.1 GBq and 1.85 GBq of RAI. In a similar setting, Musso L et al. [63] described a probability of xerostomia of about 5.26% with a median duration of 3.5 months in patients treated with a dose not exceeding 50 mCi, An YS [62] reported a probability of sialadenitis of about 5.26% in patients treated with 30 mCi and Lin WY [64] suggested a probability of dry mouth of 5.35% with rapid resolution in most cases in patients treated with a dose of 40 mCi. These studies documented the very low probability of salivary gland impairment using an ablative dose of RAI in DTC treatment.

Raza H et al. [46] documented by salivary gland scintigraphy that patients treated with less than 150 mCi showed just a small reduction in the maximum secretion and uptake rate of both the parotid and submandibular glands, with no statistical difference with patients not treated with RAI. Similar and better results were found by Wu JQ et al. [49] who analyzed salivary gland scintigraphy in 368 patients with DTC, 194 treated with RAI and 178 untreated with RAI: they found that 78 patients in the treatment group with a dose of less than 150 mCi showed no difference in the maximum glandular secretion rate and the uptake indexes of both the parotid and submandibular glands in comparison to patients in the control group, and only 5 of 78 patients had a mild sensation of dry mouth. Furthermore, an interesting prospective study published by Baudin C et al. [61], which evaluated the possible impact of RAI therapy on the salivary glands 6 months after treatment, confirmed a dose dependency of the occurrence of dry mouth sensation and stimulated salivary flow (−0.08 mL/min per 1 Gy), but without the appearance of pain or an increase in the number of patients with hyposalivation when compared to pretreatment conditions. Although some dysfunctions were noted, no obvious clinical disorders were found after 131I therapy. Summarizing, these studies quantified the possible salivary gland damage and described a very low impact of a single RAI treatment.

Le Roux MK et al. [65] presented a study considering patients treated with adjuvant doses of RAI (3.7 GBq): 162 patients were enrolled and all the symptoms of salivary gland impairment significantly decreased in number after 6 years, with the exception of the xerostomia which was also the most frequent (39.4% of the patients after RAI, 31.9% of the treated patients at the end of the follow-up). Moreover, Grewal RK et al. [66] describe a significant decrease in the number of salivary gland side effects during follow-up of patients treated with RAI (39% of patients after treatment, 5% of patients after 7 years of follow-up). Again, 14% of the patients treated with a low dose (30 mCi) and 40% of the patients treated with 75 mCi or more developed these transient problems.

These studies described a transient nature of most of the symptoms associated with salivary gland involvement, but at the same time underline that high RAI activity could induce the presentation of some problems, especially xerostomia. The main limitations of these studies were the small sample size in some cases, the retrospective nature of most of them and the different methods used to assess salivary gland impairment.

### 5.2. High-Dose Therapy and Multiple Treatments

Doses of RAI equal to or higher than 3.7 GBq, especially in multiple treatments, are clearly associated with the onset of salivary gland side effects with an incidence higher than 30% in cases of doses higher than 3.7 GBq and over 50% in patients receiving more than 7.4 Gbq [42,47]. The aforementioned paper by Musso L et al. [63] described a possible increase in the probability of salivary gland impairment with higher doses of RAI. Some other articles and reviews confirmed these findings and described that salivary gland involvement was also frequently bilateral, parotid gland involvement was most common, the probability of recovery was low and complete impairment seemed possible when more than 500 mCi (18.5 GBq) was administered [67,68,69,70,71,72,73].

In particular, Alexander C et al. [67] reported an incidence of sialadenitis of 33% in 203 patients treated with RAI at doses equal to or greater than 3.7 GBq. In particular, sialadenitis and dry mouth occurred in less than 10% and 25% of the patients treated with doses between 3.7 and 5 GBq, respectively; in contrast, more than 45% and 60% of patients treated with doses between 10 and 18.5 GBq, respectively, developed these symptoms. Dingle IF et al. [68] reported that sialadenitis was 2.47 times more likely to occur in patients who received doses greater than 150 mCi when compared with those who received less than 150 mCi. Furthermore, Klein Hesselink EN et al. [69] performed a prospective study reporting a decrease of at least 25% in stimulated whole saliva flow rate in 34% of patients treated with RAI doses ranging from 3.7 to 5.55 GBq and a ≥50% drop in saliva in 10% of patients treated with higher activity. Similar results were reported by Lee NH et al. [70], who described a probability of sialadenitis of 8.7% in patients treated with 30 mCi, 46.7% in subjects treated with 100 mCi and 69.4% in patients treated with 150 mCi. Again, considering single studies, Jeong SY et al. [71] reported a probability of salivary gland impairment and xerostomia, respectively, of 20 and 16% in patients treated with a single RAI therapy using an activity ranging from 3.7 to 5.55 GBq. This probability was higher in patients treated with higher activity. Considering these studies, the same limitations reported in studies performed on patients undergoing remnant ablation and adjuvant treatment were evident.

A relatively old review of Mandel SJ et al. [72] reported a probability of abnormal salivary gland function in as many as 80% of the patients treated with dosages of 500 mCi (18.5 GBq), approaching 100% when higher doses were used. Another review by Van Nostrand et al. [73] described an incidence of sialadenitis ranging from 2 to 67%, but excluding the study with the smaller sample, this range was restricted to 2 to 39%. The studies reported in this review, although generally old, confirmed the relationship between the dose and number of RAI treatments and the likelihood of developing salivary gland side effects. Finally, an interesting systematic review by Clement SC et al. [74], which included the study of Jeong SY et al. [71], reported that the incidence of symptomatic salivary gland dysfunction ranged from 16 to 54% of patients treated with RAI, with a general association with the dose administered and, in one study, with the number of treatments. Unfortunately, only two trials included in the review reported the mean or median dose administered, both of which were significantly higher than 3.7 Gbq. Table 2 summarizes the main results of studies with complete information.

Regarding the administration of high and/or multiple doses of RAI, it is fundamental to establish the setting to have a clear evaluation: as previously reported, a second dose of RAI could actually only be considered in the presence of biochemical or structural persistence of DTC disease, and only patients with metastatic disease require multiple RAI treatments [11]. Patients with DTC could have a real impact on their life expectancy through RAI treatment [75,76], and second-line therapies, such as tyrosine kinase inhibitors (TKIs), also present possible serious side effects [77,78] and they appear as a “one-way street” therapy without the possibility of suspension [79].

The variable rates of xerostomia/sialadenitis are also probably due to differences in study design, RAI dosing protocols and patient populations. Future research is also desirable to highlight and clarify the remaining gaps regarding long-term salivary function after RAI and the comparative effectiveness of protective agents.

## 6. Prevention of Salivary Glands Disfunction

Salivary gland protection during radioiodine therapy for DTC has become an important concern due to the potential for long-term damage. Several strategies have been explored to mitigate the negative effects of radioiodine on salivary glands, such as the use of antioxidants, pharmaceuticals and non-pharmacological methods.

### 6.1. Vitamin C and Lemon Juice

Vitamin C and lemon juice have shown promise in reducing the absorbed dose of radioiodine in the salivary glands, with studies suggesting that their administration can minimize damage and improve glandular function after treatment [80,81,82]. Similarly, the optimal timing of vitamin C and lemon juice administration has been investigated to determine the best approach to reduce salivary gland dysfunction: in particular, Liu Y et al. [80] described an optimal timing to maximize beneficial effects with administration starting 2 h after RAI, whereas Nakada et al. [82] reported 24 h after RAI as the optimal timing for lemon candy administration.

Regarding salivary gland exposure, a study by Liu B et al. [83] described only a limited effect of vitamin C, whereas Kulkarni K et al. [84] and Van Nostrand et al. [85] reported a significant effect of lemon juice in reducing the dose absorbed in the salivary glands, especially with repeated administration. In general, the administration of lemon juice is considered effective in reducing parotid gland damage and is good practice in patients treated with 131I for DTC [60].

### 6.2. Amifostine

The radioprotective agent amifostine has also been studied for its potential role in salivary gland protection in patients undergoing high-dose radioiodine therapy for DTC [86,87,88], with contrasting results. In particular, Bohuslavizki KH et al. [87] found positive results in a double-blind, placebo-controlled study, with a reduction in glandular damage. In contrast, Kim SJ et al. [88], who quantitatively evaluated the effect of amifostine use by salivary scanning, did not report any improvement in salivary gland preservation.

### 6.3. Pilocarpine

Pilocarpine, a cholinergic agent, has also been tested for its potential ability to restore salivary function by stimulating glandular activity and reducing the dose rate of radioiodine to the glands [89,90]. As for amifostine, the results were inconclusive: the study by Haghighatafshar M et al. [89] did not show a different dose rate between patients treated or not treated with pilocarpine; instead, Aframian DJ et al. [90] found that it may be beneficial in the case of impaired salivary function in patients treated with radioiodine.

### 6.4. Mechanical Interventions

Mechanical interventions have been explored as adjunctive methods to further protect the glands: in this setting, massage of the parotid glands, as reported by Hong CM et al. [91], could significantly reduce the dose rate to the glandular tissue and thus its potential damage.

### 6.5. Vitamin E

Vitamin E has been shown to play a possible role in mitigating the damage to salivary glands caused by RAI therapy. Upadhyaya et al. [92] conducted a randomized controlled trial that highlighted a beneficial impact of vitamin E supplementation in preserving salivary gland function following radioiodine therapy, noting improvements in glandular secretion and a decrease in xerostomia. Similarly, Fallahi et al. [93] reported that vitamin E provided protective effects against 131I radiation, suggesting a potential therapeutic strategy to prevent or reduce salivary gland dysfunction in this setting. These findings collectively support the inclusion of vitamin E as a possible adjunctive treatment in the management of RAI-induced salivary gland damage.

In addition, at least two interesting systematic reviews on possible salivary gland protective agents are available in the literature [94,95]: Firstly, Christou A et al. [94], which included References [82,83,84], reported the results of studies without pharmacological intervention, describing good results with sialoanalogues such as lemon juice or candy and vitamin E, and the same positive correlation with salivary gland massage. The administration of vitamin C appeared to have a limited protective effect and chewing gum was ineffective. Moreover, Auttara-Atthakorn A et al. [95] conducted a systematic review of randomized controlled trials which confirmed that not only parotid gland massage and vitamin E, but also aromatherapy, selenium, amifostine and bethanechol, may be beneficial in minimizing RAI-induced salivary gland dysfunction in patients with DTC. Table 3 summarizes the principal characteristics of the studies reported in this section.

**Figure 1:**
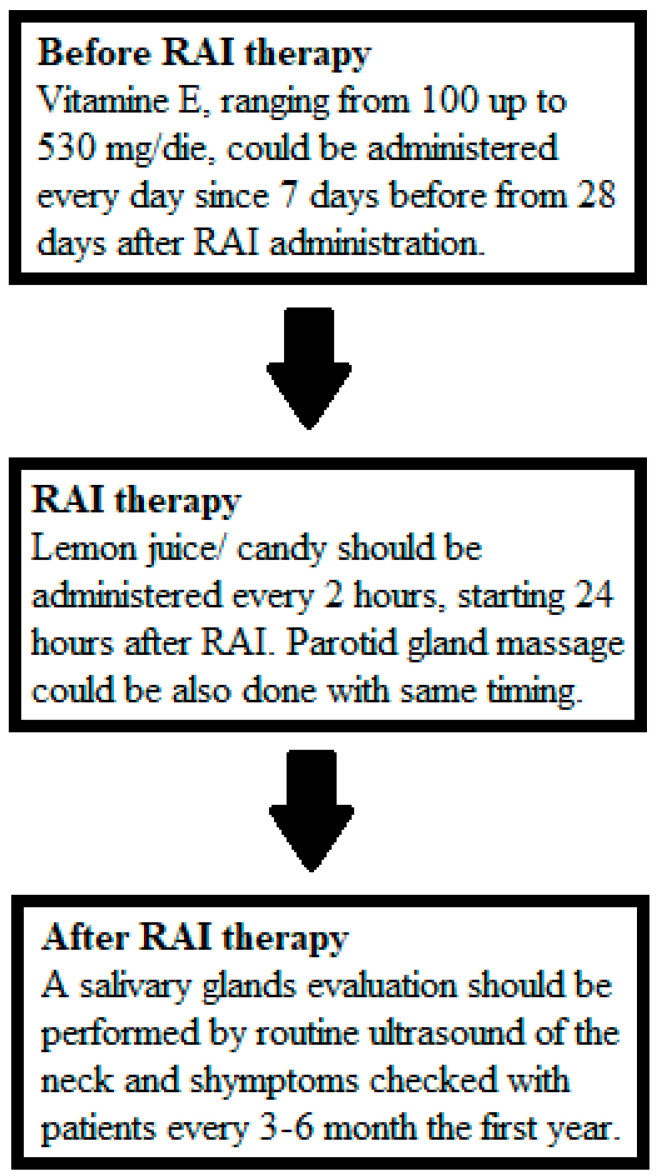
Possible clinical flow chart for the prevention and treatment of salivary gland side effects due to 131I exposure. We favor indications derived from prospective studies, studies with a larger sample size and, in other cases, indications confirmed by at least two studies. We also chose ultrasound imaging because it is already included in the usual follow-up of DTC and it does not involve other radiation exposure and/or other additional examinations.

Collectively, these studies highlight the importance of different protective strategies to minimize the risk of salivary gland damage and improve the quality of life of DTC patients undergoing radioiodine therapy. The mixed results for all the methods warrant a clearer hierarchy of evidence. The strongest evidence of efficacy is provided by lemon juice and sialoanalogues, which are therefore strongly recommended; conversely, amifostine shows limited or conflicting evidence, necessitating further studies. From a practical point of view, the recommendation could be to suggest lemon juice, vitamin E and gland massage as first-line measures due to their safety with minimal side effects and accessibility. The ATA guidelines for DTC management [11] report in recommendation 85 that no prevention strategies appear to be clearly associated with benefit and therefore do not recommend them, particularly for lemon juice, as despite clear evidence of its efficacy, the problem of the timing and frequency of administration was reported. Furthermore, the EANM guidelines for the management of DTC [60] suggest the administration of lemon juice and/or sour candy without a clear indication of timing and frequency. Parotid massage and vitamin E were not included in these guidelines. By reviewing all the reported studies and prioritizing them in the clinical workflow, we try to propose a possible simple flowchart for the prevention and monitoring of salivary gland side effects, which is subsequently shown in Figure 1.

## 7. Conclusions

Salivary gland damage, coupled with the subsequent occurrence of xerostomia and sialadenitis, is a potential adverse effect associated with RAI therapy in thyroid disease. However, low doses of RAI present a very low risk of these side effects, and when high doses are administered, the clinical rationale may justify the risk of salivary gland damage. Furthermore, available evidences suggest that measures exist to prevent gland impairment. However, for high-dose RAI in metastatic DTC, a strong emphasis on shared decision making in a dedicated tumor board is desirable, weighing survival benefits against quality-of-life impacts and aligning with modern patient-centered care. In this setting, clinicians should engage patients in shared decision making, explicitly addressing salivary risks versus oncologic benefits and prophylactic measures must be prioritized. In conclusion, while the potential for a decline in quality of life exists, it does not appear to clearly influence the indication for RAI in most cases.

## Figures and Tables

**Table 1:** A summary of the potential and limitations of each of the main methods of diagnosing and quantifying salivary gland impairment after RAI.

Assessment Methods	Potential	Limitation	Routinary Application
Questionnaire	Administration easiness	Subjective perception of patients	Possible
Ultrasound	No radiation exposure, routinely performed in the DTC follow-up.	Possibility of false positive result (no clinical manifestation)	Yes
Salivary Gland Scintigraphy	Possibility of quantifying the reduction in functionality of a single salivary gland.	Possibility of false positive result (no clinical manifestation), radiation exposure	No
MRI, CT	/	Costs, radiation exposure (CT), availability (MRI)	No

**Table 2:** Summary of the main characteristics of the reported studies on possible salivary gland impairment after RAI administration.

References	Authors	RAI Dose (GBq)	Number of Patients	Incidence of Side Effects (%)	Study Design
[42]	Horvath E et al.	1.1–1.85	156	0 **	Retrospective
[62]	An YS et al.	1.1	19	5.26 ****	Retrospective
[63]	Musso L et al.	1.1–1.85	66	0 *	Retrospective
[70]	Lee HN et al.	1.1	46	8.7 ****	Retrospective
[64]	Lin WY	1.48	56	5.35 *	Retrospective
[42]	Horvath E et al.	3.7	219	21 **	Retrospective
[62]	An YS et al.	3.7	44	38.6 ****	Retrospective
[70]	Lee HN et al.	3.7	45	46.7 ****	Retrospective
[71]	Jeong SY et al.	3.7	Nd	7.8 ***	Retrospective
[42]	Horvath E et al.	5.55	177	46.9 **	Retrospective
[62]	An YS et al.	5.55	49	34.7 ****	Retrospective
[63]	Musso L et al.	2.96–5.55	76	5.26 *	Retrospective
[67]	Alexander C et al.	4.4–5	38	20–25 ***	Retrospective
[68]	Dingle IF et al.	1.1–5.55	58	11.9 ****	Retrospective
[69]	Klein Hesselink EN et al.	3.7–5.55	56	34 *****	Prospective
[70]	Lee HN et al.	5.55	62	69.4 ****	Retrospective
[71]	Jeong SY et al.	5.55	Nd	17.9 ***	Retrospective
[68]	Dingle IF et al.	>5.55	58	23.8 ****	Retrospective
[62]	An YS et al.	7.4	3	66.7 ****	Retrospective
[70]	Lee HN et al.	7.4	4	50 ****	Retrospective

* xerostomia, ** salivary gland alteration detected by ultrasound, *** dry mouth, **** sialadenitis, ***** reduction > 25% in saliva flow rate.

**Table 3:** Summary of the main characteristics of the reported studies on possible prevention strategies for salivary gland impairment after RAI administration.

References	Authors	Prevention Agent	Number of Patients	Efficacy	Study Desing
[80]	Liu Y et al.	Vitamin C	460	Yes *	Retrospective
[81]	Jafari E et al.	Vitamin C	58	Yes	Prospective randomized
[82]	Nakada K et al.	Lemon candy	230	Yes **	Prospective
[83]	Liu B et al.	Vitamin C	72	Limited	Prospective randomized
[84]	Kulkarni K et al.	Lemon juice	9	Yes ***	Prospective
[85]	Van Nostrand et al.	Lemon juice	29	Yes ***	Retrospective
[87]	Bohuslavizki KH et al.	Amifostine	17	Yes	Retrospective
[88]	Kim SJ et al.	Amifostine	80	No	Prospective
[89]	Haghighatafshar M et al.	Pilocarpine	22	No	Prospective randomized
[90]	Aframian DJ et al.	Pilocarpine	5	Limited ****	Nd
[91]	Hong CM et al.	Parotid gland massage	44	Yes ***	Prospective
[92]	Upadhyaya et al.	Vitamin E	82	Yes	Prospective randomized
[93]	Fallahi B et al.	Vitamin E	36	Yes	Prospective randomized

* administration from 2 h after RAI, ** administration from 24 h after RAI, *** reduction in salivary gland irradiation, **** the study reported some benefit in the administration after RAI damage.

## References

[1] Miller E.H., Soley M.H. (1948). Treatment of Graves’ disease with radioiodine. Am. J. Med..

[2] Sun Y., Zhao Y., Sun D., Mu X., Li J., Lu C., Lu L., Lin C., Lv J., Li R. (2025). Reflective analysis on the current 131I adjuvant therapy indications in intermediate- and high-risk differentiated thyroid cancer. Eur. J. Nucl. Med. Mol. Imaging.

[3] Corvilain B., Hamy A., Brunaud L., Borson-Chazot F., Orgiazzi J., Bensalem Hachmi L., Semrouni M., Rodien P., Lussey-Lepoutre C. (2018). Treatment of adult Graves’ disease. Ann. Endocrinol..

[4] Bellini P., Dondi F., Zilioli V., Gatta E., Cavadini M., Cappelli C., Viganò G.L., Bertagna F. (2024). The Role of Radioiodine Therapy in Differentiated Thyroid Cancer Arising from Struma Ovarii: A Systematic Review. J. Clin. Med..

[5] Bernard D., Desruet M.D., Wolf M., Roux J., Boin C., Mazet R., Gallazzini C., Calizzano A., Vuillez J.P., Allenet B. (2014). Radioiodine therapy in benign thyroid disorders. Evaluation of French nuclear medicine practices. Ann. Endocrinol..

[6] Miller C., Al-Jabri A., O’Murchada L., Mustafa M., Cooke J., Phelan N., Healy M.L. (2024). Use of a dosimetry-based RAI protocol for treatment of benign hyperthyroidism optimises response while minimising exposure to ionising radiation. Clin. Endocrinol..

[7] Metso S., Jaatinen P., Huhtala H., Luukkaala T., Oksala H., Salmi J. (2004). Long-term follow-up study of radioiodine treatment of hyperthyroidism. Clin. Endocrinol..

[8] Stachura A., Gryn T., Kałuża B., Budlewski T., Franek E. (2020). Predictors of euthyreosis in hyperthyroid patients treated with radioiodine 131I-: A retrospective study. BMC Endocr. Disord..

[9] Campennì A., Siracusa M., Ruggeri R.M. (2024). Oldie but Goldie: The Fundamental Role of Radioiodine in the Management of Thyroid Cancer. J. Clin. Med..

[10] Dueñas-Disotuar S., Piñar-Gutiérrez A., de Lara-Rodríguez I., Sastre-Marcos J., Anda-Apiñániz E., Oleaga-Alday A., Galofré-Ferrater J.C., Orois-Añon A., Alcázar-Lázaro V., Martínez-Guasch L. (2024). Bone metastasis in differentiated thyroid cancer: Spanish multicenter study of clinical characteristics, survival and prognostic factors. Front. Endocrinol..

[11] Haugen B.R., Alexander E.K., Bible K.C., Doherty G.M., Mandel S.J., Nikiforov Y.E., Pacini F., Randolph G.W., Sawka A.M., Schlumberger M. (2016). 2015 American Thyroid Association Management Guidelines for Adult Patients with Thyroid Nodules and Differentiated Thyroid Cancer: The American Thyroid Association Guidelines Task Force on Thyroid Nodules and Differentiated Thyroid Cancer. Thyroid.

[12] Albano D., Dondi F., Bellini P., Bertagna F., Giovanella L. (2023). Biomarkers and Molecular Imaging in Postoperative DTC Management. Integrated Diagnostics and Theranostics of Thyroid Diseases.

[13] Bellini P., Dondi F., Gatta E., Zilioli V., Albano D., Cappelli C., Bertagna F. (2024). Prognostic role and characteristics of the indeterminate response in differentiated thyroid cancer: A systematic review. Endocrine.

[14] Albano D., Bellini P., Dondi F., Calabrò A., Casella C., Taboni S., Lombardi D., Treglia G., Bertagna F. (2023). Temporal Evolution and Prognostic Role of Indeterminate Response Sub-Groups in Patients with Differentiated Thyroid Cancer after Initial Therapy with Radioiodine. Cancers.

[15] Durante C., Haddy N., Baudin E., Leboulleux S., Hartl D., Travagli J.P., Caillou B., Ricard M., Lumbroso J.D., De Vathaire F. (2006). Long-term outcome of 444 patients with distant metastases from papillary and follicular thyroid carcinoma: Benefits and limits of radioiodine therapy. J. Clin. Endocrinol. Metab..

[16] Fard-Esfahani A., Emami-Ardekani A., Fallahi B., Fard-Esfahani P., Beiki D., Hassanzadeh- Rad A., Eftekhari M. (2014). Adverse effects of radioactive iodine-131 treatment for differentiated thyroid carcinoma. Nucl. Med. Commun..

[17] Wu W., Li S., Xu K., Meng Z. (2023). Hazard ratios of second primary malignancy after radioiodine for differentiated thyroid carcinoma: A large-cohort retrospective study. Endokrynol. Pol..

[18] Terrazas J.R., Marins C.R.P., Correa M.E.P., Assumpção L.V.M.D., Zantut-Wittmann D.E. (2025). Influence of Radioiodine Therapy on Oral Health and Salivary Production in Patients With Differentiated Thyroid Carcinoma. Endocr. Pract..

[19] Elisei R., Vivaldi A., Pacini F. (2003). Biology and clinical application of the NIS gene. Tumori.

[20] Dohán O., De la Vieja A., Paroder V., Riedel C., Artani M., Reed M., Ginter C.S., Carrasco N. (2003). The sodium/iodide Symporter (NIS): Characterization, regulation, and medical significance. Endocr. Rev..

[21] Bruno R., Giannasio P., Ronga G., Baudin E., Travagli J.P., Russo D., Filetti S., Schlumberger M. (2004). Sodium iodide symporter expression and radioiodine distribution in extrathyroidal tissues. J. Endocrinol. Investig..

[22] Zanzonico P.B. (1997). Radiation dose to patients and relatives incident to 131I therapy. Thyroid.

[23] Taprogge J., Abreu C., Vávrová L., Carnegie-Peake L., Rushforth D., Gape P., Gear J., Murray I., Wong K.H., Newbold K. (2023). Initial results of the INSPIRE clinical trial- investigating radiation dosimetry for differentiated thyroid cancer patients. Front. Nucl. Med..

[24] Melo D.R., Brill A.B., Zanzonico P., Vicini P., Moroz B., Kwon D., Lamart S., Brenner A., Bouville A., Simon S.L. (2015). Organ Dose Estimates for Hyperthyroid Patients Treated with (131)I: An Update of the Thyrotoxicosis Follow-Up Study. Radiat. Res..

[25] Liu B., Huang R., Kuang A., Zhao Z., Zeng Y., Wang J., Tian R. (2011). Iodine kinetics and dosimetry in the salivary glands during repeated courses of radioiodine therapy for thyroid cancer. Med. Phys..

[26] Abuqbeitah M., Demir M., Sağer S., Asa S., Işıkcı N.I., Sönmezoğlu K. (2023). SPECT/CT-based dosimetry of salivary glands and iodine-avid lesions following 131I therapy. Health Technol..

[27] Sánchez Barrueco A., González Galán F., Alcalá Rueda I., Santillán Coello J.M., Barrio Dorado M.P., Villacampa Aubá J.M., Escanciano Escanciano M., Llanos Jiménez L., Mahillo Fernández I., Cenjor Español C. (2020). Incidence and risk factors for radioactive iodine-induced sialadenitis. Acta Otolaryngol..

[28] Sadiç M., Korkmaz M., Gültekin S.S., Demircan K. (2016). Alterations in ADAMTS12 gene expression in salivary glands of radioiodine-131-administered rats. Nucl. Med. Commun..

[29] Sadic M., Demirel K., Halacli S.O., Karakok E., Koca G., Ekinci O., Demircan K., Korkmaz M. (2018). Expression of ADAMTS2 and ADAMTS5 in the salivary gland of rats after radioiodine therapy. Nucl. Med. Commun..

[30] Turgut B., Ozdemir O., Erselcan T. (2006). Evaluation of the p53 tumor suppressor gene mutation in normal rat salivary gland tissue after radioiodine application: An experimental study. Adv. Ther..

[31] Kim J.M., Choi M.E., Kim S.K., Kim J.W., Kim Y.M., Choi J.S. (2020). Keratinocyte Growth Factor-1 Protects Radioiodine-Induced Salivary Gland Dysfunction in Mice. Int. J. Environ. Res. Public Health.

[32] Jung J.H., Kim J.H., Jung M.H., Kim S.W., Jeong B.K., Woo S.H. (2020). Protective Effect of Alpha-Lipoic Acid on Salivary Dysfunction in a Mouse Model of Radioiodine Therapy-Induced Sialoadenitis. Int. J. Mol. Sci..

[33] Kim J.W., Kim J.M., Kim S.K., Kim Y.M., Choi J.S. (2018). Protective Effect of Ginseng on Salivary Dysfunction Following Radioiodine Therapy in a Mouse Model. Thyroid.

[34] Kim J.M., Kim J.W., Choi M.E., Kim S.K., Kim Y.M., Choi J.S. (2019). Protective effects of curcumin on radioiodine-induced salivary gland dysfunction in mice. J. Tissue Eng. Regen. Med..

[35] Bohuslavizki K.H., Klutmann S., Jenicke L., Kröger S., Buchert R., Mester J., Clausen M. (1999). Salivary gland protection by S-2-(3-aminopropylamino)-ethylphosphorothioic acid (amifostine) in high- dose radioiodine treatment: Results obtained in a rabbit animal model and in a double-blind multi-arm trial. Cancer Biother. Radiopharm..

[36] Tsur N., Avishai G., Alkan U., Hod R., Shpitzer T., Bitton E., Gilat H. (2023). Ultrasonographic Features of Salivary Glands after Radioiodine Therapy in Patients with Thyroid Cancer. Laryngoscope.

[37] Simões Lima G.A., López R.V.M., de Freitas R.M.C., Willegaignon J., Sapienza M.T., Chammas M.C., Coura-Filho G.B. (2020). Evaluation of Parotid Salivary Gland Echo Texture by Ultrasound Examinations and Correlation With Whole-Body Scintigraphy After Radioiodine Therapy in Patients with Differentiated Thyroid Carcinoma. J. Ultrasound Med..

[38] Christou A., Papastavrou E., Merkouris A., Charalambous A. (2021). A pretest-posttest pilot study for the development and preliminary validation of a tool for the clinical assessment of radioiodine induced sialadenitis. SAGE Open Med..

[39] Buchholzer S., Thakachy Subha S., Tchérémissinoff L., Boselie F., Triponez F., Faure F., Lopez J.M., Borner U., Kleinjung T., Seebach J.D. (2021). The RAI-6 Questionnaire: A New Screening Questionnaire to Monitor Complications of Radioiodine Treatment. Front. Surg..

[40] Buchholzer S., Faure F., Tcheremissinoff L., Herrmann F.R., Lombardi T., Ng S.K., Lopez J.M., Borner U., Witt R.L., Irvine R. (2022). Novel Multidisciplinary Salivary Gland Society (MSGS) Questionnaire: An International Consensus. Laryngoscope.

[41] Moreddu E., Baumstarck-Barrau K., Gabriel S., Fakhry N., Sebag F., Mundler O., Chossegros C., Taïeb D. (2017). Incidence of salivary side effects after radioiodine treatment using a new specifically- designed questionnaire. Br. J. Oral Maxillofac. Surg..

[42] Horvath E., Skoknic V., Majlis S., Tala H., Silva C., Castillo E., Whittle C., Niedmann J.P., González P. (2020). Radioiodine-Induced Salivary Gland Damage Detected by Ultrasonography in Patients Treated for Papillary Thyroid Cancer: Radioactive Iodine Activity and Risk. Thyroid..

[43] Kim D.W. (2015). Ultrasonographic Features of the Major Salivary Glands after Radioactive Iodine Ablation in Patients with Papillary Thyroid Carcinoma. Ultrasound Med. Biol..

[44] Brozzi F., Rago T., Bencivelli W., Bianchi F., Santini P., Vitti P., Pinchera A., Ceccarelli C. (2013). Salivary glands ultrasound examination after radioiodine-131 treatment for differentiated thyroid cancer. J. Endocrinol. Investig..

[45] Lima G.A.S., López R.V.M., Ozório G.A., de Freitas R.M.C., Willegaignon J., Sapienza M.T., Chammas M.C., Coura-Filho G.B. (2020). Ultrasonography Echotexture as a surrogate for Sialadenitis secondary to 131I Radioiodine Therapy for differentiated Thyroid Cancer: A review and meta-analysis. Clinics.

[46] Raza H., Khan A.U., Hameed A., Khan A. (2006). Quantitative evaluation of salivary gland dysfunction after radioiodine therapy using salivary gland scintigraphy. Nucl. Med. Commun..

[47] Bohuslavizki K.H., Brenner W., Lassmann S., Tinnemeyer S., Tönshoff G., Sippel C., Wolf H., Clausen M., Henze E. (1996). Quantitative salivary gland scintigraphy in the diagnosis of parenchymal damage after treatment with radioiodine. Nucl. Med. Commun..

[48] Malpani B.L., Samuel A.M., Ray S. (1996). Quantification of salivary gland function in thyroid cancer patients treated with radioiodine. Int. J. Radiat. Oncol. Biol. Phys..

[49] Wu J.Q., Feng H.J., Ouyang W., Sun Y.G., Chen P., Wang J., Xian J.L., Huang L.H. (2015). Systematic evaluation of salivary gland damage following I-131 therapy in differentiated thyroid cancer patients by quantitative scintigraphy and clinical follow-up. Nucl. Med. Commun..

[50] Shen J., Xu X.Q., Su G.Y., Hu H., Shi H.B., Liu W., Wu F.Y. (2018). Intravoxel incoherent motion magnetic resonance imaging of the normal-appearing parotid glands in patients with differentiated thyroid cancer after radioiodine therapy. Acta Radiol..

[51] Nabaa B., Takahashi K., Sasaki T., Okizaki A., Aburano T. (2012). Assessment of salivary gland dysfunction after radioiodine therapy for thyroid carcinoma using non-contrast-enhanced CT: The significance of changes in volume and attenuation of the glands. AJNR Am. J. Neuroradiol..

[52] Campennì A., Avram A.M., Verburg F.A., Iakovou I., Hänscheid H., de Keizer B., Petranović Ovčariček P., Giovanella L. (2023). The EANM guideline on radioiodine therapy of benign thyroid disease. Eur. J. Nucl. Med. Mol. Imaging.

[53] Madu N.M., Skinner C., Oyibo S.O. (2022). Cure Rates After a Single Dose of Radioactive Iodine to Treat Hyperthyroidism: The Fixed-Dose Regimen. Cureus.

[54] Ross D.S., Burch H.B., Cooper D.S., Greenlee M.C., Laurberg P., Maia A.L., Rivkees S.A., Samuels M., Sosa J.A., Stan M.N. (2016). 2016 American Thyroid Association Guidelines for Diagnosis and Management of Hyperthyroidism and Other Causes of Thyrotoxicosis. Thyroid.

[55] Maia A.L., Scheffel R.S., Meyer E.L., Mazeto G.M., Carvalho G.A., Graf H., Vaisman M., Maciel L.M., Ramos H.E., Tincani A.J. (2013). The Brazilian consensus for the diagnosis and treatment of hyperthyroidism: Recommendations by the Thyroid Department of the Brazilian Society of Endocrinology and Metabolism. Arq. Bras. Endocrinol. Metabol..

[56] Chielens L., Nauwynck E., Bourgeois S., Staels W., Vanbesien J., Gies I., Ernst C., Everaert H., De Schepper J. (2024). A Belgian single centre outcome study of radioiodine treatment in adolescents with Graves’ disease. Sci. Rep..

[57] Okkalides D. (2016). Thyroid Patient Salivary Radioiodine Transit and Dysfunction Assessment Using Chewing Gums. Cancer Biother. Radiopharm..

[58] Ford H., Johnson L., Purdie G., Feek C. (1997). Effects of hyperthyroidism and radioactive iodine given to ablate the thyroid on the composition of whole stimulated saliva. Clin. Endocrinol..

[59] Sunavala-Dossabhoy G. (2018). Radioactive iodine: An unappreciated threat to salivary gland function. Oral Dis..

[60] Luster M., Clarke S.E., Dietlein M., Lassmann M., Lind P., Oyen W.J., Tennvall J., Bombardieri E., European Association of Nuclear Medicine (EANM) (2008). Guidelines for radioiodine therapy of differentiated thyroid cancer. Eur. J. Nucl. Med. Mol. Imaging..

[61] Baudin C., Bressand A., Buffet C., Menegaux F., Soret M., Lê A.T., Cardon T., Broggio D., Bassinet C., Huet C. (2023). Dysfunction of the Salivary and Lacrimal Glands After Radioiodine Therapy for Thyroid Cancer: Results of the START Study After 6-Months of Follow-Up. Thyroid.

[62] An Y.S., Yoon J.K., Lee S.J., Song H.S., Yoon S.H., Jo K.S. (2013). Symptomatic late-onset sialadenitis after radioiodine therapy in thyroid cancer. Ann. Nucl. Med..

[63] Musso L., Maltese C., Beretta G., Patelli I., Raffa S., Piccardo A., Fiz F., Vera L., Albertelli M., Minuto M. (2025). Low-Dose Versus Standard-Dose Radioiodine Therapy in Differentiated Thyroid Cancer: Focus on Tolerability in a Retrospective Evaluation. Pharmaceuticals.

[64] Lin W.Y., Shen Y.Y., Wang S.J. (1996). Short-term hazards of low-dose radioiodine ablation therapy in postsurgical thyroid cancer patients. Clin. Nucl. Med..

[65] Le Roux M.K., Graillon N., Guyot L., Taieb D., Galli P., Godio-Raboutet Y., Chossegros C., Foletti J.M. (2020). Salivary side effects after radioiodine treatment for differentiated papillary thyroid carcinoma: Long-term study. Head Neck.

[66] Grewal R.K., Larson S.M., Pentlow C.E., Pentlow K.S., Gonen M., Qualey R., Natbony L., Tuttle R.M. (2009). Salivary gland side effects commonly develop several weeks after initial radioactive iodine ablation. J. Nucl. Med..

[67] Alexander C., Bader J.B., Schaefer A., Finke C., Kirsch C.M. (1998). Intermediate and long-term side effects of high-dose radioiodine therapy for thyroid carcinoma. J. Nucl. Med..

[68] Dingle I.F., Mishoe A.E., Nguyen S.A., Overton L.J., Gillespie M.B. (2013). Salivary morbidity and quality of life following radioactive iodine for well-differentiated thyroid cancer. Otolaryngol. Head Neck Surg..

[69] Klein Hesselink E.N., Brouwers A.H., de Jong J.R., van der Horst-Schrivers A.N., Coppes R.P., Lefrandt J.D., Jager P.L., Vissink A., Links T.P. (2016). Effects of Radioiodine Treatment on Salivary Gland Function in Patients with Differentiated Thyroid Carcinoma: A Prospective Study. J. Nucl. Med..

[70] Lee H.N., An J.Y., Lee K.M., Kim E.J., Choi W.S., Kim D.Y. (2015). Salivary gland dysfunction after radioactive iodine (I-131) therapy in patients following total thyroidectomy: Emphasis on radioactive iodine therapy dose. Clin. Imaging.

[71] Jeong S.Y., Kim H.W., Lee S.W., Ahn B.C., Lee J. (2013). Salivary gland function 5 years after radioactive iodine ablation in patients with differentiated thyroid cancer: Direct comparison of pre- and postablation scintigraphies and their relation to xerostomia symptoms. Thyroid.

[72] Mandel S.J., Mandel L. (2003). Radioactive iodine and the salivary glands. Thyroid.

[73] Van Nostrand D. (2011). Sialoadenitis secondary to ¹³¹I therapy for well-differentiated thyroid cancer. Oral Dis..

[74] Clement S.C., Peeters R.P., Ronckers C.M., Links T.P., van den Heuvel-Eibrink M.M., Nieveen van Dijkum E.J., van Rijn R.R., van der Pal H.J., Neggers S.J., Kremer L.C. (2015). Intermediate and long-term adverse effects of radioiodine therapy for differentiated thyroid carcinoma--a systematic review. Cancer Treat. Rev..

[75] Mazzaferri E.L. (2000). Long-term outcome of patients with differentiated thyroid carcinoma: Effect of therapy. Endocr. Pract..

[76] Tuttle R.M., Ahuja S., Avram A.M., Bernet V.J., Bourguet P., Daniels G.H., Dillehay G., Draganescu C., Flux G., Führer D. (2019). Controversies, Consensus, and Collaboration in the Use of 131I Therapy in Differentiated Thyroid Cancer: A Joint Statement from the American Thyroid Association, the European Association of Nuclear Medicine, the Society of Nuclear Medicine and Molecular Imaging, and the European Thyroid Association. Thyroid.

[77] Miyamoto S., Tsukaguchi A., Kuhara H., Otsuki T., Shiroyama T., Tamiya M., Tamiya A., Nishino K., Takeda Y., Kijima T. (2025). Real-World Efficacy and Safety of Lenvatinib in Advanced or Recurrent Thymic Carcinoma: A Multicenter Retrospective Study in Japan. Thorac. Cancer.

[78] Bhat G., Chanthar V.K.M.M., Rahalkar A., Sooraj R., Philip R.C., Mayilvaganan S., Singh K.R., Chand G., Ramakant P., Mishra A. (2025). Efficacy and Safety of Tyrosine Kinase Inhibitors in Downstaging and Palliation in Patients with Advanced Differentiated Thyroid Cancer—A Multicentre study. Indian. J. Surg. Oncol..

[79] Goto H., Kiyota N., Otsuki N., Imamura Y., Chayahara N., Suto H., Nagatani Y., Toyoda M., Mukohara T., Nibu K.I. (2018). Successful treatment switch from lenvatinib to sorafenib in a patient with radioactive iodine- refractory differentiated thyroid cancer intolerant to lenvatinib due to severe proteinuria. Auris Nasus Larynx.

[80] Liu Y., Wang Y., Zhang W. (2022). Optimal administration time of vitamin C after 131I therapy in differentiated thyroid cancer based on propensity score matching. Front. Surg..

[81] Jafari E., Alavi M., Zal F. (2018). The evaluation of protective and mitigating effects of vitamin C against side effects induced by radioiodine therapy. Radiat. Environ. Biophys..

[82] Nakada K., Ishibashi T., Takei T., Hirata K., Shinohara K., Katoh S., Zhao S., Tamaki N., Noguchi Y., Noguchi S. (2005). Does lemon candy decrease salivary gland damage after radioiodine therapy for thyroid cancer?. J. Nucl. Med..

[83] Liu B., Kuang A., Huang R., Zhao Z., Zeng Y., Wang J., Tian R. (2010). Influence of vitamin C on salivary absorbed dose of 131I in thyroid cancer patients: A prospective, randomized, single-blind, controlled trial. J. Nucl. Med..

[84] Kulkarni K., Van Nostrand D., Atkins F., Mete M., Wexler J., Wartofsky L. (2014). Does lemon juice increase radioiodine reaccumulation within the parotid glands more than if lemon juice is not administered?. Nucl. Med. Commun..

[85] Van Nostrand D., Bandaru V., Chennupati S., Wexler J., Kulkarni K., Atkins F., Mete M., Gadwale G. (2010). Radiopharmacokinetics of radioiodine in the parotid glands after the administration of lemon juice. Thyroid.

[86] Bohuslavizki K.H., Klutmann S., Brenner W., Mester J., Henze E., Clausen M. (1998). Salivary gland protection by amifostine in high-dose radioiodine treatment: Results of a double-blind placebo- controlled study. J. Clin. Oncol..

[87] Bohuslavizki K.H., Klutmann S., Bleckmann C., Brenner W., Lassmann S., Mester J., Henze E., Clausen M. (1999). Salivary gland protection by amifostine in high-dose radioiodine therapy of differentiated thyroid cancer. Strahlenther. Onkol..

[88] Kim S.J., Choi H.Y., Kim I.J., Kim Y.K., Jun S., Nam H.Y., Kim J.S. (2008). Limited cytoprotective effects of amifostine in high-dose radioactive iodine 131-treated well-differentiated thyroid cancer patients: Analysis of quantitative salivary scan. Thyroid.

[89] Haghighatafshar M., Ghaedian M., Etemadi Z., Entezarmahdi S.M., Ghaedian T. (2018). Pilocarpine effect on dose rate of salivary gland in differentiated thyroid carcinoma patients treated with radioiodine. Nucl. Med. Commun..

[90] Aframian D.J., Helcer M., Livni D., Markitziu A. (2006). Pilocarpine for the treatment of salivary glands’ impairment caused by radioiodine therapy for thyroid cancer. Oral Dis..

[91] Hong C.M., Son S.H., Kim C.Y., Kim D.H., Jeong S.Y., Lee S.W., Lee J., Ahn B.C. (2014). Emptying effect of massage on parotid gland radioiodine content. Nucl. Med. Commun..

[92] Upadhyaya A., Zhou P., Meng Z., Wang P., Zhang G., Jia Q., Tan J., Li X., Hu T., Liu N. (2017). Radioprotective effect of vitamin E on salivary glands after radioiodine therapy for differentiated thyroid cancer: A randomized-controlled trial. Nucl. Med. Commun..

[93] Fallahi B., Beiki D., Abedi S.M., Saghari M., Fard-Esfahani A., Akhzari F., Mokarami B., Eftekhari M. (2013). Does vitamin E protect salivary glands from I-131 radiation damage in patients with thyroid cancer?. Nucl. Med. Commun..

[94] Christou A., Papastavrou E., Merkouris A., Frangos S., Tamana P., Charalambous A. (2016). Clinical Studies of Nonpharmacological Methods to Minimize Salivary Gland Damage after Radioiodine Therapy of Differentiated Thyroid Carcinoma: Systematic Review. Evid. Based Complement. Altern. Med..

[95] Auttara-Atthakorn A., Sungmala J., Anothaisintawee T., Reutrakul S., Sriphrapradang C. (2022). Prevention of salivary gland dysfunction in patients treated with radioiodine for differentiated thyroid cancer: A systematic review of randomized controlled trials. Front. Endocrinol..

